# Mechanical bone growth stimulation by magnetic fibre networks obtained through a competent finite element technique

**DOI:** 10.1038/s41598-017-07731-6

**Published:** 2017-09-11

**Authors:** Wolfram A. Bosbach

**Affiliations:** University of Cambridge, Engineering Department, Cambridge, CB2 1PZ UK

## Abstract

Fibre networks combined with a matrix material in their void phase make the design of novel and smart composite materials possible. Their application is of great interest in the field of advanced paper or as bioactive tissue engineering scaffolds. In the present study, we analyse the mechanical interaction between metallic fibre networks under magnetic actuation and a matrix material. Experimentally validated FE models are combined for that purpose in one joint simulation. High performance computing facilities are used. The resulting strain in the composite’s matrix is not uniform across the sample volume. Instead we show that boundary conditions and proximity to the fibre structure strongly influence the local strain magnitude. An analytical model of local strain magnitude is derived. The strain magnitude of 0.001 which is of particular interest for bone growth stimulation is achievable by this assembly. In light of these findings, the investigated composite structure is suitable for creating and for regulating contactless a stress field which is to be imposed on the matrix material. Topics for future research will be the advanced modelling of the biological components and the potential medical utilisation.

## Introduction

Materials incorporating a fibre structure have been an essential constituent in various fields of application for decades^1,2^. Today, a range of several distinguishable categories of fibre materials exists: polymeric non-woven fabrics^3,4^, actin networks and cytoskeletons in the field of biomaterials^5,6^, or also theoretical computer generated network materials^7–9^. The mechanical behaviour^9–12^, the thermal conductivity^9,13^, or the magnetic response^14,15^ are amongst the investigated physical properties.

The purpose of this present study is to investigate the suitability of a specific fibre-matrix composite material for its suitability as mechanically active tissue engineering scaffold. This fibre material is manufactured by sintering randomly stacked stainless steel fibres^16–19^. Its basic properties have been investigated by several studies. It has been shown that beam theory offers an elegant simplification for numerical studies about its mechanics. Further it is known about this material that greater fibre volume fraction *f* reduces inside the material the average length *λ* of fibre segments between sintered inter-fibre bonds (Table 1). This has a strong effect on the local mechanical response of the material which is dominated by fibre deflection over fibre elongation^12^. The deformation under magnetic actuation of individual fibres has been investigated^15^. This study now extends the scope of the work to entire network geometries. Numerical studies offer a very useful tool in this context. The highly delicate geometry of this network material can be studied numerically at a great level of detail. Local behaviour of the material can be quantified. Those local results would equally be obtainable from experimental measurements only under a great expense of resources. Whenever available, the corresponding experimental measurements allow the calibration of the numerical models.

**Table 1:** Network samples.

Sample	FVF	Segment length		Meshed elements				Additional constrains	
	*f* [%]	*λ* [μm]		Network nodes ${\hat{{\boldsymbol{N}}}}^{i}$	B31/B32	CONN3D2		Singularities	Zero-Pivots
Sample-10%	10	237		4,006	4,002	221		5	1
Sample-15%	15	186		7,156	7,262	693		4	—
Sample-20%	20	153		7,826	7,963	863		8	—
**Sample**	**FVF**	**Network nodes per 2D pixel slice on 25** ^**3**^ **-grid**					**Relative standard deviation**		
	***f*** **[%]**	**Mean**	**Median x-axis**		**Median y-axis**	**Median z-axis**	**x-axis [%]**	**y-axis [%]**	**z-axis [%]**
Sample-10%	10	160.2	164		165	158	11.4	35.5	38.4
Sample-15%	15	286.2	286		282	291	16.8	15.7	15.9
Sample-20%	20	313.0	308		313	300	13.1	11.6	20.5

Average fibre segment length *λ* of the material^17^, number of meshed elements, additional constraints, and distribution of network nodes.

Unlike previous work about this specific fibre material, the present study combines it with a matrix material to a fibre-matrix composite. Static, conventional applications of fibre-matrix composites have been proposed for composites of bioglass^20,21^ or cordierite^22,23^. In the field of biomedical engineering, metallic^24,25^ and since then also non-metallic^26,27^ fibre networks have been tested for their ability to improve bone cement mechanics. The exhibited properties document very clearly improvements from the composite structure for this orthopaedic application. In non-static or smart applications of fibre-matrix composites, material properties are amended during the application process. Magnetic nanoparticles combined with cellulose fibres are in the discussion for the design of data storage applications and magnetographic printing or filtering^14,28^. This work has been extended also to incorporate bacterial cellulose^29,30^. In a different context, magnetic fibre networks could be applied to design heat exchangers of variable drag^31,32^. The assembly of this present study has been discussed for its suitability as scaffold in tissue engineering^33^. In this case, the purpose of the fibres would be to deliver under magnetic actuation a mechanical stimulus for the enhanced growth of bone cells. Various other fibre network materials are also investigated for their application as tissue scaffolds^29,34^. The delivery of a mechanical stimulus from scaffolds for influencing cells and their development is an on-going research topic on tissue level and on organ level^35–37^. For bone cells, it is known that cyclical loadings at 1 Hz of the strain magnitude 0.001 are beneficial for the tissue’s remodelling behaviour^38^. A beneficial effect of a stand-alone magnetic field on bone growth is equally known^39,40^.

Yet, the mechanical interaction between fibre phase and matrix phase in fibre-matrix composites is so far only insufficiently understood. The purpose of the present study is to make a contribution to this field and to investigate the mechanical response of a matrix inserted in the void phase of a fibre network under magnetic actuation. Previous work has already analysed for this specific material simplified single-fibre geometries under magnetic actuation^15^. Or global values have been predicted analytically for fibre assemblies^41,42^. This present study investigates complete fibre network geometries and analyses the matrix strain on local level. The results of this study are obtained by means of experimentally validated finite element (FE) models and lead to an analytical model for the local matrix strain in cube shaped fibre networks under magnetic actuation. The presented results were part of a dissertation project at the University of Cambridge^19^.

## Mathematical notation

The following notation is used in this document. Scalars are given as *x*, vectors as $\bar{x}$, and 2^*nd*^ rank tensors as $\bar{\bar{x}}$. The vector product “×” and dot product “·” are applied. Relations which are greater-than and approximately equal are indicated as “≳”. All fibre network nodes *n* of one sample *i* form the node set *N* ^*i*^. The total number of nodes in that sample *i* is defined as ${\hat{N}}^{i}$. Equality between two volumes is written “ ⇔ ”, a volume containing a volume subset is given as “⊂”. The operator for geometry definition by the intersection of two bodies is “∩”. 3D spheres are specified for midpoint and radius as *O*_(*midpoint*,*R*)_. Their respective boundary is written *o*_(*midpoint*,*R*)_ = ∂*O*_(*midpoint*,*R*)_, geometric cube faces are written *F*.

## Modelling Methods and Material

**Figure 1:**
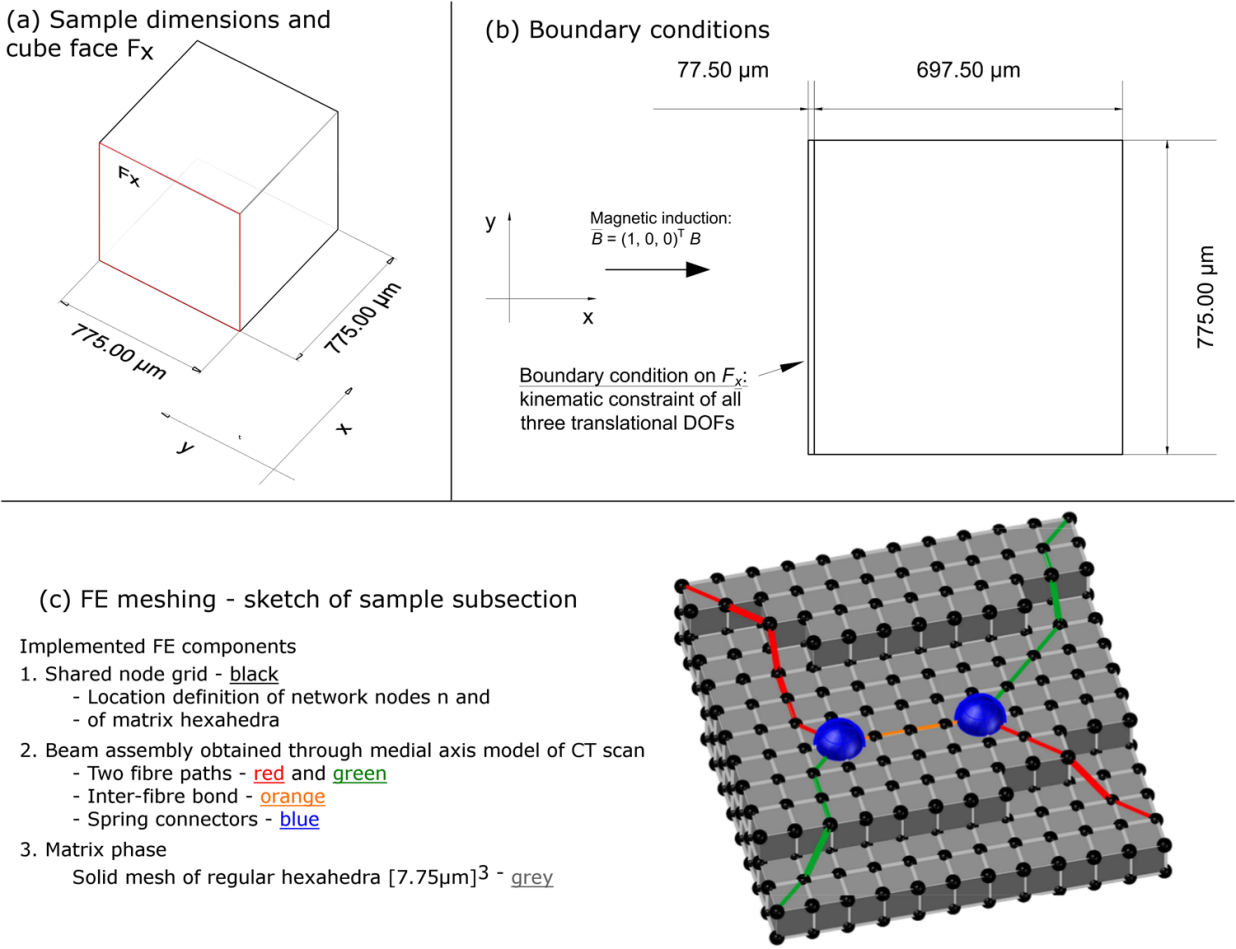
FE model: (**a**) Sample dimensions with cube face *F*_*x*_, (**b**) boundary conditions, (**c**) FE mesh.

For the investigations of the present study, a FE model (Fig. 1) of linear elasticity is developed. It describes the matrix strain under magnetic actuation in interaction with the fibre material. The quantitative evaluation of local fibre network density and local strain fields follows the scheme of Eqs 10 to 13 and leads to the analytical model presented by this study (Eq. 16).

### Material samples and fibre network geometry extraction

FE modelling of the fibre network follows steps of an approach based on beam theory^12^. Three cube shaped fibre network samples are used in this present study (Fig. 1a and Table 1). This allows the investigation of three different values of fibre volume fraction *f*: 10, 15, and 20%. The influence of *f* on the mechanical response is of particular interest.

These networks are produced by N.V. Bekaert S.A. (Belgium) from American Iron And Steel Institute (AISI) 316 L (*d* ≈ 40 μm) or by Nikko Techno Ltd. (Japan) from AISI 444 steel fibres, assembled as fibre stacks, compressed, and sintered to fibre mats. During the sintering step, inter-fibre bonds form. The sample sections are cut by electronic discharge machining from the sintered mats and computed tomography (CT) scans are acquired by General Electric (Germany) for a resolution of *res* = 7.75 μm. Due to the available computing capacities, the sample cube volume is set to *V* = (775.00 μm)^3^. In the CT scans, this sample cube side length is the equivalent of 100 pixels.

A skeletonisation algorithm^43–45^ is applied and returns as output the medial axis paths of the fibre bodies contained in the CT data. Resulting from the chosen CT scan resolution *res* = 7.75 μm, the location of all medial axes is defined inside the sample cubes on a [7.75 μm · 100]^3^-grid. Nodes defining network medial axis location are referred to as network nodes in the following. Their total number per sample ${\hat{N}}^{i}$ is known to increase for greater *f* ^17,18^ and is given in Table 1 for the three network samples.

### FE meshing

While previous studies have simulated fibre networks for a void inter-fibre space^9,12^, the present study combines a fibre phase *V*_*f*_ and a matrix phase *V*_*M*_ in the sample volume *V*. The meshing of both phases and the defined interaction rules are discussed in the following (Fig. 1c). The mesh is implemented for the FE solver Abaqus 6.13^46^ (Tables 2 and 3). By their definition in the present study, *V*_*M*_ equals *V* while *V*_*f*_ is only a subset:

1 $$V\iff {V}_{M}\subset {V}_{f}$$

**Table 2:** Implemented FE types.

Component	Element Type	Identifier	Geometry	Interpolation/Connection
Fibre	Timoshenko beam	B31	3D	linear interpolation
	Timoshenko beam	B32	3D	quadratic interpolation
Inter-fibre bond	Spring connector	CONN3D2	3D	join & torsional spring
Matrix	Hexahedron	C3D8R	3D	reduced integration

Abaqus^46^ element types used in model implementation.

**Table 3:** FE modelling parameters.

Model component	Description	Parameter	Dimensional value
CT scan	Resolution	*res*	7.75 μm/pixel
	Sample side length	*L*	775.00 μm = 100 pixel
	Sample volume	*V*	(775.00 μm)^3^
Fibre network	Fibre volume fraction	*f*	10, 15, 20%
	Diameter	*d*	40 μm
	Joint stiffness	*K*_*joint*_	= *s* *E*_*f*_ *A*_*f*_
	Scaling factor	*s*	5 μm^12^
	Fibre stiffness	*E*_*f*_	200 GPa^54^
	Poisson’s ratio	*ν*	0.3
Matrix	Hexahedron side length	*L*_*hex*_	7.75 μm = 1 pixel
	Hexahedron volume	*V*_*hex*_	(7.75 μm)^3^
	Number of hexahedra	*N*_*hex*_	100^3^
	Matrix stiffness	*E*_*m*_	Collagenous bone^47^: 200 KPa
			Granulation tissue^48^: 1 MPa
			Immature bone^49^: 0.1 GPa
	Poisson’s ratio	*ν*	0.3
Magnetic actuation and BC	Ferromagnetic response	${\bar{\tau }}_{M}$	$=\,{A}_{f}{L}_{f}(\overline{{M}_{s}}\times \overline{B})$
	Magnetic saturation	*M*_*S*_	1.6 MA/m^15^
	Magnetic induction	*B*	0.25–2.00T
	BC depth	*h*_*BC*_	77.50 μm^12^

Abaqus^46^ element types, fibre/matrix material, and BC used for model implementation.

Eq. 1 simplifies the volume description by disregarding the marginal volume of fibres whose medial axis is located at a distance to the surface *S* of less than *d*/2.

#### Matrix phase

A mesh of regular hexahadra elements (C3D8R) fills *V*. These hexahedra represent *V*_*M*_. For simplifying the interaction with the fibre bodies, the hexahedra side length *L*_*hex*_ is set to the CT scan resolution *res* = 7.75 μm = 1 pixel. The number of hexahedra in *V* follows as *N*_*hex*_ = 100^3^, their volume as *V*_*hex*_ = (7.75 μm)^3^.

The values of matrix stiffness *E*_*M*_ are chosen as it would be applicable for three stages in bone tissue development: collagenous bone^47^, granulation tissue^48^, and immature bone^49^. The value for immature bone was chosen from the early phase of bone development. The value for collagenous bone is slightly greater than found in the literature for enhancing the FE solver convergence.

#### Beam assembly

The fibre medial axes are transferred in the present study into beam assemblies. Two Timoshenko beams^50–52^ are implemented. Results for linear interpolation (B31) and quadratic interpolation (B32) are analysed and compared to predictions from previous work^12^. The beams are simulated for a simplified round cross section of diameter *d* = 40 μm (i.e. *A*_*f*_ = (*d*/2)^2^ *π*), a Young’s modulus of *E*_*f*_ = 200 GPa, and for a Poisson’s ratio of *ν* = 0.3. The sintered inter-fibre joints are implemented by torsional springs (CONN3D2). Their stiffness is simulated as *K*_*joint*_ = *sE*_*f*_ *A*_*f*_ with *s* = 5 μm. This value of *s* has been found to a pproximate results from experimental measurements^12,17^. The number of network nodes in Table 1 refers to exclusively those nodes which define beam start points and end points. The implementation for the FE solver requires additional technical nodes. Those purely technical nodes provide additional integration points for B32 elements, indicate the normal relative to the beam axis, and one is needed for each CONN3D2 element.

#### Interaction rules

The implementation of the interaction between *V*_*fibre*_ and *V*_*matrix*_ (Fig. 1c) is based on a previously published model for osteosynthesis applications^53^. The regular hexahedra and the beam mesh share the 100^3^-node grid which is defined by the CT scan pixels. Three main simplifications are made in the modelling:

- *V*_*f*_ and *V*_*M*_ overlap, a double section assignment exists for up to *max*(*f*) = 20% of *V*.
- The interaction between both phases is reduced to the shared node grid.
- The mechanical interaction is set to identical displacement $\bar{u}$ at each node.

This means that more complex mechanical interaction as it appears in biological tissue between cells and scaffold is not considered at this point. These simplifications are made while, as in the original model, the following holds:

2 $${E}_{f}\gg {E}_{M}$$

### FE simulation and BC

In the case of a void inter-fibre space, the Cauchy stress tensor $\bar{\bar{\sigma }}$ is defined for a fibre network consisting of *V*_*f*_ and the void matrix phase *V*_*void*_^9^:

3 $$\bar{\bar{\sigma }}\ne 0\,\forall \bar{x}\in {V}_{f},\bar{\bar{\sigma }}=0\,\forall \bar{x}\in {V}_{void}$$

In this study, the relationship is modified for *V*_*M*_. Eq. 4 defines now the stress fields in the fibre-composite as:

4 $$\bar{\bar{\sigma }}\ne 0\,\forall \bar{x}\in {V}_{f},\bar{\bar{\sigma }}\ne 0\,\forall \bar{x}\in {V}_{M}$$

This relationship might seem obvious. It is mentioned explicitly at this point because Eq. 4 is required for the evaluation of imposed matrix strain with regard to the magnitude of *E*_*M*_ and with regard to the respective magnetic actuation.

#### Magnetic actuation and BC

In the simulations, a magnetic induction vector $\bar{B}$ is imposed (Fig. 1b). The ferromagnetic mechanical response of the fibres is modelled as moment vector ${\bar{\tau }}_{B}$. It is calculated for each beam element of length *L*_*f*_ under the assumption of complete magnetisation parallel to the beam axis^15^:

5 $${\bar{\tau }}_{B}={A}_{f}{L}_{f}(\overline{{M}_{s}}\times \overline{B})$$

In Eq. 9, ${\overline{\tau }}_{M}$ of all fibres in the respective sample is added into the equilibrium condition of moments. The actuation model is applied for *M*_*s*_ = 1.6 MA/m. This value of *M*_*s*_ has been experimentally validated for ferritic AISI 444^15^. The geometries of the network samples were obtained for austenitic AISI 316L. As part of this study’s modelling assumptions, the network is simulated for the hypothetical *M*_*s*_ of AISI 444. It has been shown that the geometry of the AISI 316L networks can similarly also be produced by AISI 444 fibres^17,18^. The imposed magnetic induction vector $$\bar{B}=[\begin{array}{c}1\\ 0\\ 0\end{array}]B$$ is orientated along the x-axis. At the time of this study’s design, experiments were carried out at the University of Cambridge in which this type of in-plane actuation was tested.

The sample surface *S* of the sample volume *V* is defined by six quadratic cube faces, two of them perpendicular each to one of the three geometric axes. The cube face *F*_*x*_, marked in red in Fig. 1a, is used for the definition of the boundary conditions (BC). All three translational degrees of freedom (DOF) of beam elements located on the cube face *F*_*x*_ are kinematically constrained up to a depth of *h*_*BC*_ = 77.50 μm into the material. This value of *h*_*BC*_ was applied as it provides a match to the experimental stiffness magnitude^12,17^.

#### Equilibrium conditions

The FE solver is run under the assumption of linear elasticity and for the equilibrium conditions of forces (Eq. 6) and moments (Eq. 8). It is required that $div(\bar{\bar{\sigma }})=0$ is fulfilled. Body force per volume $\overline{f}$ is set to 0 in the present study; gravitational forces being a typical example for $\overline{f}$. Together with the surface traction vector $\bar{t}$, it defines the equilibrium of forces. Surface traction itself is defined by $\bar{\bar{\sigma }}$ and the outer-normal vector: $\bar{t}=\bar{\bar{\sigma }}\,\cdot \,{\bar{n}}_{out}$ ^55^.

6 $${\int }_{S}\bar{t}\,dS+{\int }_{V}\overline{f}\,dV=0$$

As the magnetic response is modelled as moment vector ${\overline{\tau }}_{B}$, the general condition for equilibrium of forces can be simplified for the present study. Considering that $\overline{f}\mathrm{=}\,0$ it follows that:

7 $${\rm{Eq}}{\rm{.}}\,{\rm{6}}\mathop{\Rightarrow }\limits^{\bar{f}\mathrm{=}\,0}{\int }_{{F}_{x}}\bar{t}\,dS=0$$

Relative to the origin of point vector $\bar{x}$, the general form of the equilibrium of moments^55^ is defined:

8 $${\int }_{S}(\bar{x}\times \bar{t})\,dS+{\int }_{V}(\bar{x}\times \bar{f})\,dV=0$$

The simplification of Eq. 6 by $\bar{f}=0$ can be repeated for Eq. 8. The sum of ${\bar{\tau }}_{B}$ which is imposed on the fibre volume *V*_*f*_ is added. The modified equation representing the present study and considering the sum of imposed ${\bar{\tau }}_{B}$ follows as:

9 $${\rm{Eq}}\,{\rm{.8}}\mathop{\Rightarrow }\limits^{\bar{f}=\,\mathrm{0,}\,+{\bar{\tau }}_{B}}\quad {\int }_{{F}_{x}}(\bar{x}\times \bar{t})\,dS\mathop{\underbrace{+\sum _{{V}_{fibre}}{\overline{\tau }}_{B}}}\limits_{{\rm{magnetic}}\,\,{\rm{actuation}}}=0$$

The Lagrangian reference frame^56^ is used for Eqs 6 to 9. The alternative Eulerian reference frame can be advantageous for the modelling of fluids^57,58^. During the study, the solver runs into up to eight singularities and one zero-pivot (Table 1). For the facilitation of the solving step, singularities and zero-pivots are constrained for their respective DOF. Due to the size of the beam mesh (>10^3^) this number of additionally constrained nodes is negligible with regard to the presented mechanical analyses.

### Network node counting and strain field averaging methods

**Figure 2:**
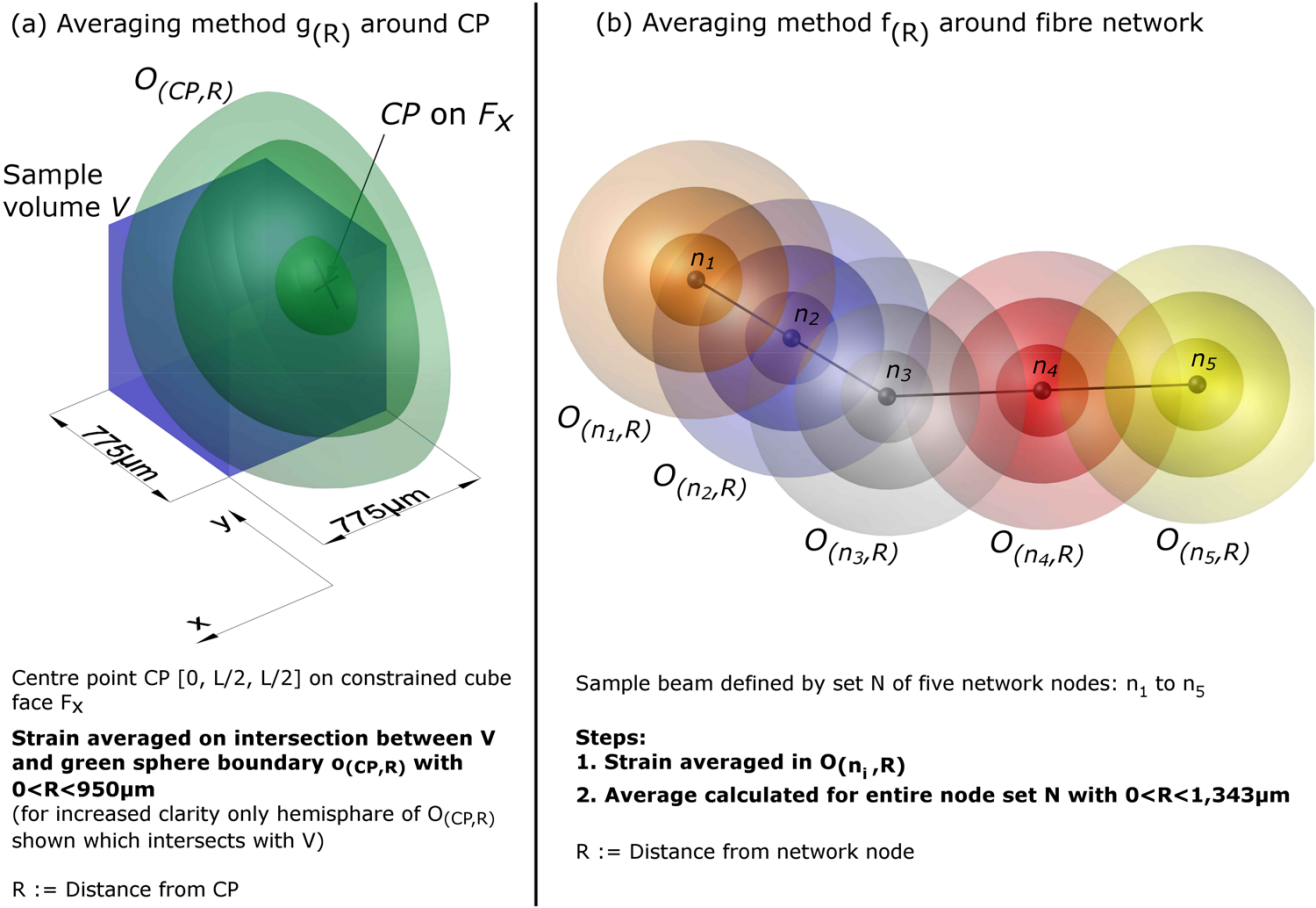
Averaging methods: (**a**) Averaging method *g*_(*R*)_ around *CP* on constrained cube face *F*_*x*_ (Eq. 12) and (**b**) *f*_(*R*)_ around fibre network (Eq. 13).

Eqs 10 and 11 are applied in this present study for further analyses of local fibre network density. For that purpose, a centre point $$CP=[\begin{array}{c}0\\ L/2\\ L/2\end{array}]=[\begin{array}{c}0\\ 387.50\\ 387.50\end{array}]$$ μm is defined on the constrained cube face *F*_*x*_ (Fig. 2a). The function *y*_(*R*)_ stands for the relative share of nodes in sample volume *V* which is located on the sphere boundary *o*_(*CP*,*R*)_ of radius *R*.

10 $${y}_{(R)}^{i}=\sum _{V\cap {o}_{(CP,R)}}{N}^{i}/{\hat{N}}^{i}$$

11 $$\begin{array}{ccc}{Y}_{(R)}^{i} & = & {\sum }_{V\cap {O}_{(CP,R)}}{N}^{i}/{\hat{N}}^{i}\\  & = & {\int }_{0}^{R}{y}_{(R)}\,dR\\ i & \in  & \{10{\rm{ \% }},15{\rm{ \% }},20{\rm{ \% }}\}:\text{samples of 10, 15, and 20\%}\,f\end{array}$$

*Y*_(*R*)_ stands for the integral of *y*_(*R*)_ over *R* and counts network nodes of *V* inside the sphere *O*_(*CP*,*R*)_. *y*_(*R*)_ and *Y*_(*R*)_ lead to *g*_(*R*)_ which is the first of two applied strain averaging methods. Both strain averaging methods, *g*_(*R*)_ and *f*_(*R*)_, quantify the local strain obtained in the matrix phase *V*_*M*_. Two equivalent strain measures, von-Mises strain measure *ε*_*v*.*M*._ and maximum principal strain *ε*_*Pmax*_^59,60^, are calculated from the 3D strain fields of *V*_*M*_. While *ε*_*v*.*M*._ is generally recommended for ductile material, *ε*_*Pmax*_ is of greater precision for brittle materials^61^. The material behaviour type of bone has been shown to depend on the deformation rate *dε*/*dt* ^62^.

*g*_(*R*)_ quantifies the matrix strain on the boundary *o*_(*CP*,*R*)_ where it intersects with *V* (Fig. 2a and Eq. 12). *f*_(*R*)_ places in a first step a sphere *O*_(*n*,*R*)_ on each matrix node *n* and averages the matrix strain within *O*_(*n*,*R*)_. In the second step, the average is obtained across the entire node set *N* ^*i*^ (Fig. 2b and Eq. 13). *g*_(*R*)_ quantifies the matrix strain depending on the distance to the constrained cube face. *f*_(*R*)_ quantifies the matrix strain depending on the average distance to the fibre network structure.

12 $${g}_{(R)}^{i,j}=\frac{1}{S}{\int }_{V\cap {o}_{(CP,R)}}{\varepsilon }_{j}\,dS$$

13 $$\begin{array}{cc}{f}_{(R)}^{i,j} & =\mathop{\underbrace{\frac{1}{{\hat{N}}^{i}}\quad \sum _{{N}^{i}}}}\limits_{2.\,{\rm{a}}{\rm{v}}{\rm{e}}{\rm{r}}{\rm{a}}{\rm{g}}{\rm{e}}\,{\rm{f}}{\rm{o}}{\rm{r}}\,{\rm{n}}{\rm{o}}{\rm{d}}{\rm{e}}\,{\rm{s}}{\rm{e}}{\rm{t}}\,{N}^{i}}\mathop{\overbrace{[\frac{1}{\frac{4}{3}\pi {R}^{3}}\,\,{\int }_{{O}_{(n,R)}}{\varepsilon }_{j}dV]}}\limits^{1.\,{\rm{a}}{\rm{v}}{\rm{e}}{\rm{r}}{\rm{a}}{\rm{g}}{\rm{e}}\,{\varepsilon }_{j}\,{\rm{i}}{\rm{n}}\,O\,{\rm{o}}{\rm{n}}\,{\rm{n}}{\rm{o}}{\rm{d}}{\rm{e}}\,n}\\ i\, & \in \{10{\rm{ \% }},15{\rm{ \% }},20{\rm{ \% }}\}:\text{samples of 10, 15, and 20\%}\,f\\ {\varepsilon }_{j}\, & \in \{{\varepsilon }_{v.M.},\,{\varepsilon }_{Pmax}\}\end{array}$$

### Hardware

For the solving step, the FE code is run on the Cambridge High Performance Computing Cluster Darwin. The cluster provides a total of 9600 cores by 600 quad server Dell C6220 chassis. Two 2.60 GHz eight core Intel Sandy Bridge E5-2670 processors with sixteen cores in total form one node with 64 GB of RAM (4 GB per core)^63^.

## Results

The matrix phase of the meshed fibre-matrix composite (Fig. 1) is analysed for its deformation. Results about deformation patterns and deformation magnitude under magnetic actuation are presented. Distinctive patterns in the local matrix strain distribution are discovered. The results lead to an analytical model for the matrix strain magnitude in cube shaped fibre-matrix composites. A particular focus is the influence of the local fibre network density.

### Local fibre network density

**Figure 3:**
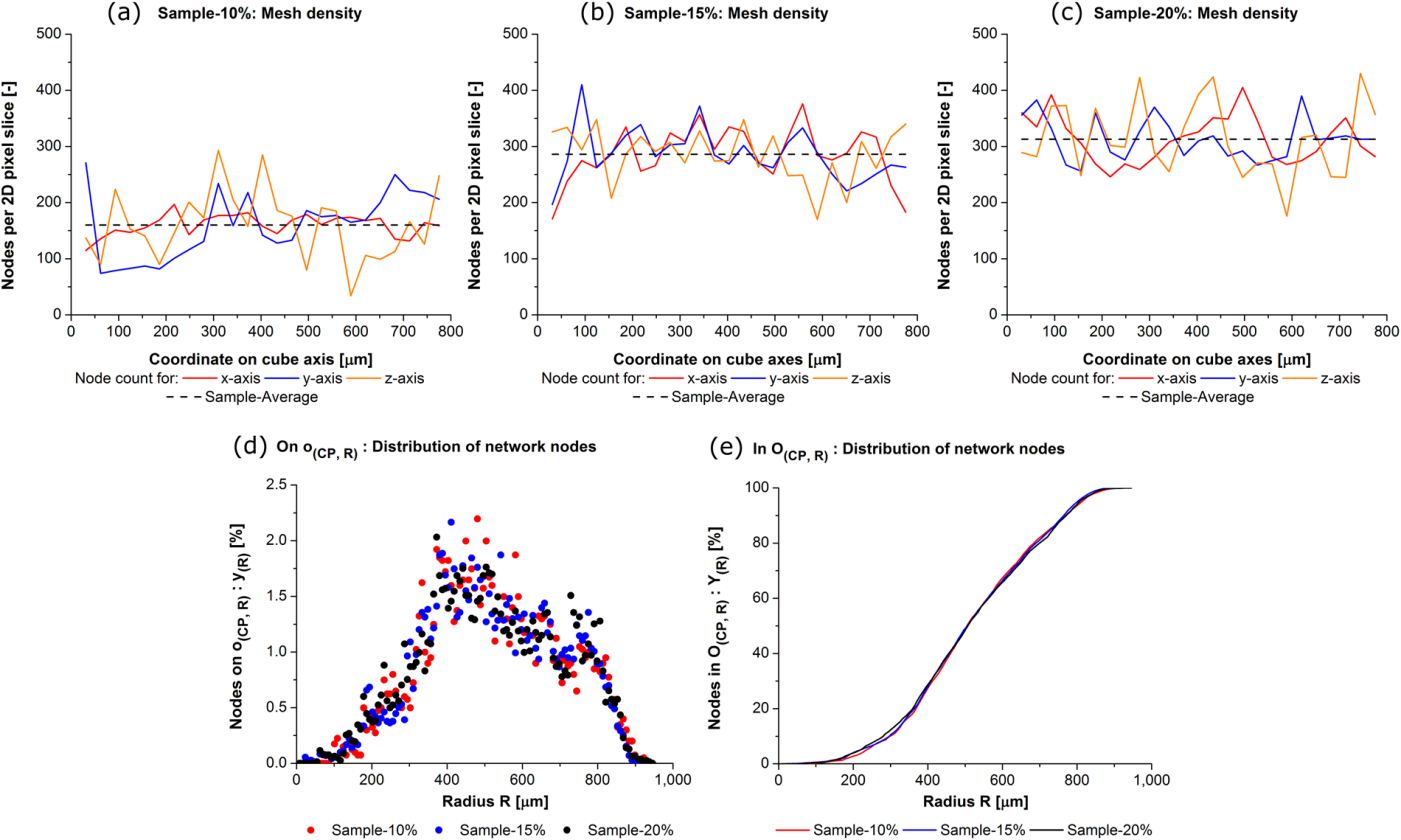
Local network density: (**a**) to (**c**) network node distribution along Cartesian axes x, y, z, and (**d**)/(**e**) network node distribution on/in sphere *O*_(*CP*,*R*)_.

As explained above, the analysed fibre material is a statistical material. This requires for mechanical modelling knowledge about the distribution of fibre density and about the distribution of fibre orientation. In particular, this knowledge is of importance for the discussion of the local deformation patterns discovered in the present study. Previous analyses have shown that the main fibre orientation direction lies in the xy-plane; in the xy-plane itself, no preferred direction of orientation can be seen^17^. Figure 3 and Table 1 of the present study analyse now further the local distribution of material density across the three samples by quantification of the network node distribution. The density of network nodes relates directly to the fibre density in *V*.

**Figure 4:**
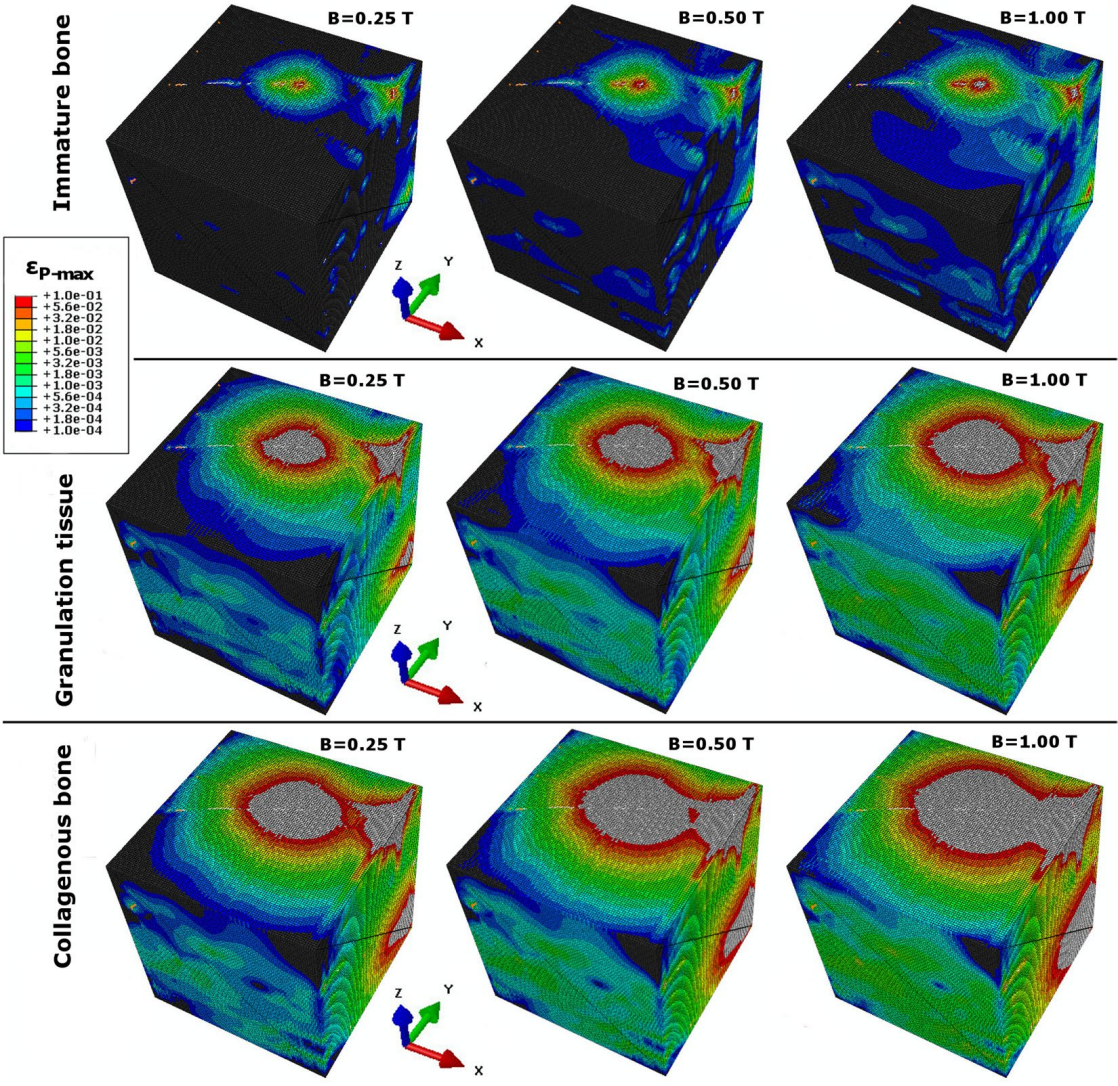
Deformation plot: *ε*_*Pmax*_ of Sample-15% for beam B31 under magnetic actuation, green range on colour bar scaled to magnitude 0.001.

**Figure 5:**
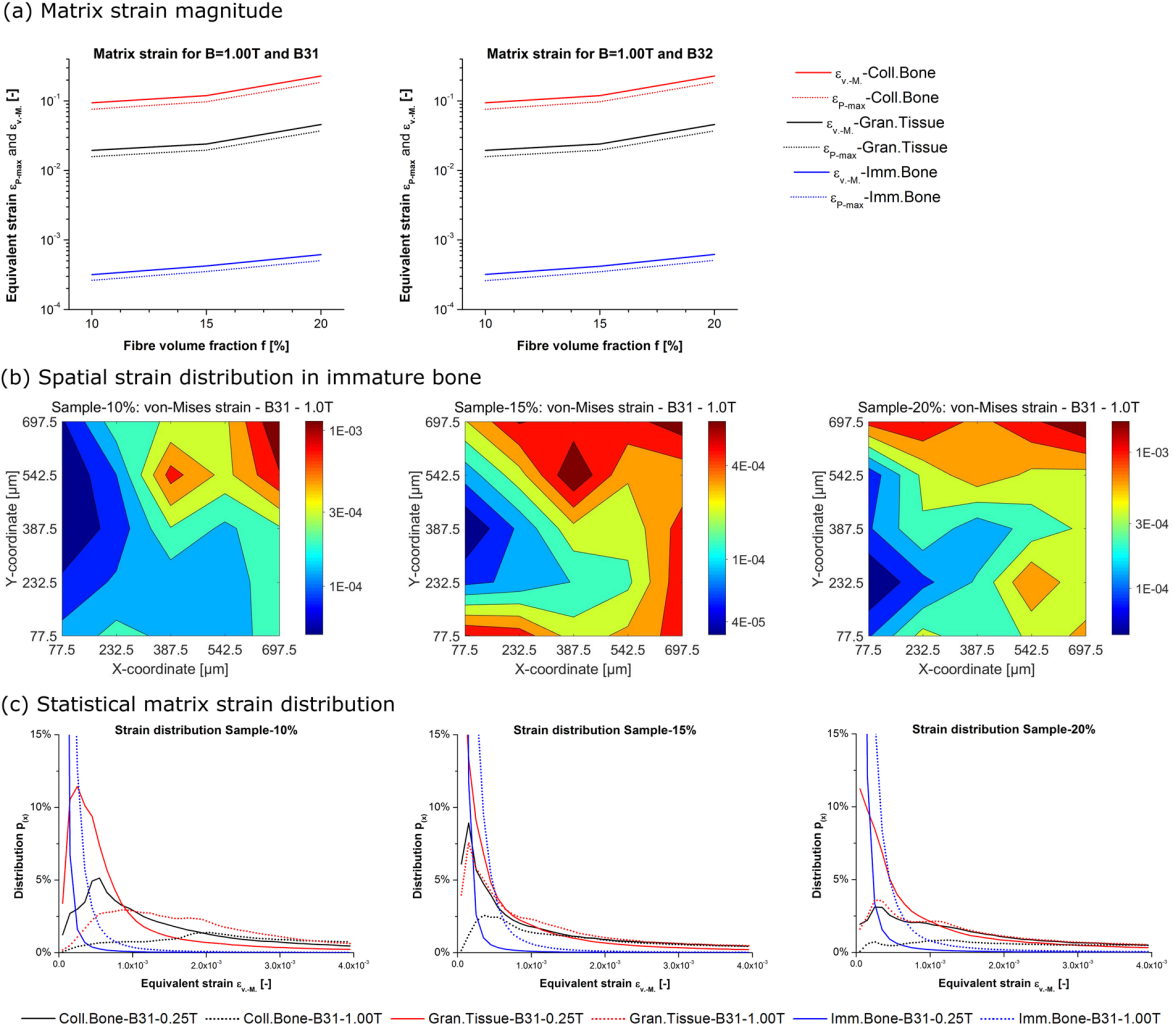
Matrix strain: (**a**) magnitude *ε*_*v*.*M*._ and *ε*_*Pmax*_ depending on *f*, (**b**) spatial distribution of *ε*_*v*.*M*._ in immature bone, (**c**) statistical *ε*_*v*.*M*._ distribution.

The main result is that the variation of node density along the three Cartesian axes is far greater than the density variation relative to the sphere *O*_(*CP*,*R*)_. The network nodes are counted per 2D pixel slice of a 25^3^-grid along each of the three Cartesian axes. Table 1 shows the obtained numerical average, the median, and the standard deviation. A visible deviation exists in each of the three samples along each Cartesian axis. The maximum standard deviation relative to the average exists in *min*(*f*) = 10% along y and z. These two findings are very interesting as their cause lies in the compression step during manufacturing. Future network design might be able to exploit this further. However, this would make necessary a degree of control about production parameters greater than the one available in today’s process. The corresponding distributions are plotted in Fig. 3a to c together with each sample’s average. These three plots do not exhibit distinct local sample sections of outstanding greater/lesser material density. However, the extent of density variation along the three Cartesian axes becomes visible in these plots. The plot of Sample-10% shows clearly the greatest density variation relative to the sample average. Since the magnetic induction is imposed on the matrix material by the fibre network (Eq. 5), the rather uneven material density distribution along the three axes leads to local variations of obtained matrix strain (Figs 4 and 5b).

The share of nodes located on/in *O*_(*CP*,*R*)_ is plotted in Fig. 3d and e as function *y*_(*R*)_ and *Y*_(*R*)_ (Eqs 10 and 11). Both, *y*_(*R*)_ and *Y*_(*R*)_, exhibit a variation of the density distribution along *R* which is of far lesser magnitude than the one along the three Cartesian axes. This holds for each of the three samples. In Fig. 3d, *y*_(*R*)_ is plotted. The three samples follow the same pattern of three distinct sections along *R* which can be seen in the distribution:

- *R* ≤ 387.5 μm: *y*_(*R*)_ increases exponentially. *O*_(*CP*,*R*)_ corresponds to the smallest sphere drawn in Fig. 2a and is entirely contained in *V*.
- 387.5 μm < *R* ≤ 775 μm: *O*_(*CP*,*R*)_ exceeds the extensions of *V* at the cube’s sides and *y*_(*R*)_ decreases, in a good approximation linearly.
- 775 μm < *R*: *O*_(*CP*,*R*)_ intersects with *V* only at the four cube corners opposite to cube face *F*_*x*_ as drawn for the greatest sphere in Fig. 2a. *y*_(*R*)_ drops sharply to 0.

**Figure 6:**
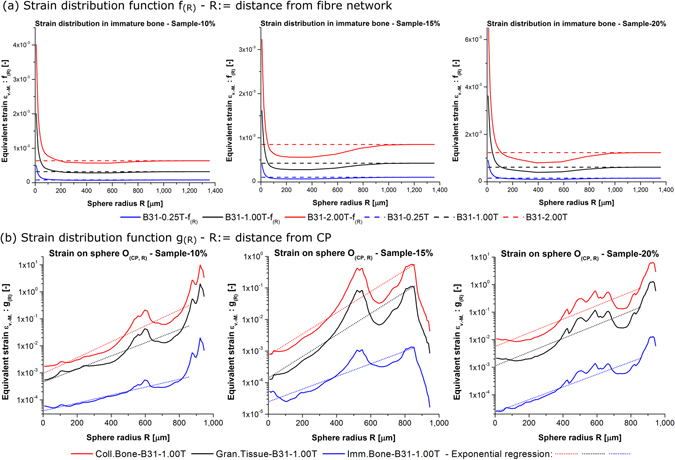
Matrix strain: (**a**) Distribution around fibre network and (**b**) around *CP* on cube face *F*_*x*_.

In Fig. 3e, it can be seen that the value of *Y*_(*R*)_ increases for greater *R* continuously at different rates. A node share of 100% is reached when the entire *V* is contained in *O*_(*CP*,*R*)_, i.e *R* > 950 μm. Very important for the context of this study, the patterns are independent of *f* (i.e. it is obtained in each of the three samples). The findings of Fig. 3d and e are new for this specific material and are used in this present study for deriving the analytical model of the matrix strain magnitude (Fig. 6b and Eq. 16).

### Matrix strain magnitude and distribution

The FE assembly is simulated for the three sample geometries. The obtained magnitude of matrix strain and its distribution inside the sample, as well as, its statistical distribution are discussed now in this section.

#### Deformation plot

Figure 4 contains the deformation plots of Sample-15% under magnetic actuation for all three matrix materials defined in Table 3. The green range on the colour bar is scaled to the magnitude 0.001 because of the particular interest with regard to applications including biomechanical bone growth stimulation^38^. Each of the nine plots contains locally this specific magnitude of matrix strain. The required magnitude of *B* is within the experimentally achievable range. Up to *B* = 1.00 can be seen as practical. This finding is of importance because it suggests the general suitability of this specific network material as scaffold for bone growth stimulation under magnetic actuation. Local maxima and minima are visible on *S*. For an increase from *B* = 0.25 to 1.00, the extension of the green areas increases, spreading further into the material. Greater *E*_*M*_ reduces them respectively. The quantitative analysis of the matrix strain magnitude and distribution follows in Fig. 5.

#### Strain magnitude

Figure 5a plots the strain magnitude when averaged across the sample volume *V* for the previously shown interesting value of *B* = 1.00. The mechanical response is scaled linearly by *B* due to the modelling assumption of linear elasticity. The values of *ε*_*Pmax*_ for brittle behaviour are marginally smaller than the values of *ε*_*v*.*M*._ for each value of *f*, and for each *E*_*M*_. This relationship is also predicted in the literature^59^ and holds independently of the Timoshenko beam (Eq. 15). Lesser *E*_*M*_ increases the matrix strain non-linearly. Of interest is the result that the average matrix strain clearly exceeds 0.001 in the case of collagenous bone and granulation tissue. At this early stage of bone tissue development, lesser values of *B* are sufficient. For immature bone and *B* = 1.00, average matrix strain between 10^−4^ and 0.001 is obtained. A greater density of fibre network material increases the matrix strain for the given size of *V*. It is known for this network material that its mechanical response is influenced at this scale by a pronounced size effect^12^. Whether and how this size effect also influences the matrix strain under magnetic actuation is at this point one very worthwhile topic of future research.

The two Timoshenko beams lead to almost identical results for the matrix strain magnitude (Table 4). B32 is known to marginally reduce the network’s mechanical stiffness^12^:

14 $${E}_{{\rm{B}}31}\gtrsim {E}_{{\rm{B}}32}$$

**Table 4:** Timoshenko beam.

Δ = *ε*_*B*32_/*ε*_*B*31_ − 1		von-Mises strain *ε*_*υ*.M._			max. principal strain *ε*_*Pmax*_		
	*B* [T]	0.50	1.00	2.00	0.50	1.00	2.00
Sample-10%	Collagenous Bone	0.00191‰	0.00192‰	0.00194‰	0.00131‰	0.00109‰	0.00121‰
	Granulation Tissue	0.01377‰	0.01371‰	0.01372‰	0.01026‰	0.01091‰	0.00998‰
	Immature Bone	0.19231‰	0.19234‰	0.19233‰	0.16799‰	0.16806‰	0.16802‰
Sample-15%	Collagenous Bone	0.00081‰	0.00082‰	0.00084‰	0.00174‰	0.00182‰	0.00187‰
	Granulation Tissue	0.01620‰	0.01616‰	0.01631‰	0.01978‰	0.01925‰	0.01988‰
	Immature Bone	0.72658‰	0.72669‰	0.72658‰	0.76742‰	0.76737‰	0.76720‰
Sample-20%	Collagenous Bone	0.00014‰	0.00013‰	0.00019‰	0.00082‰	0.00069‰	0.00099‰
	Granulation Tissue	0.00438‰	0.00441‰	0.00430‰	0.00675‰	0.00716‰	0.00688‰
	Immature Bone	0.65166‰	0.65149‰	0.65153‰	0.63249‰	0.63233‰	0.63240‰

Difference Δ of obtained matrix strain.

Conversely, under magnetic actuation the quadratic Timoshenko beam B32 leads to marginally greater matrix strain than linear B31 if *B* = *const*. This relation holds for both equivalent strain measures.

15 $$\begin{array}{cc}\text{if}\,\,B=const,\,\,\, & {E}_{M}=const,\,\,\,f=const\text{, it holds that}\\ {\varepsilon }_{Pmax} & \,\lesssim \,{\varepsilon }_{v.M.}\,\,\,\text{, valid for both Timoshenko beams (B31 or B32)}\\ {\varepsilon }_{\text{B31}} & \,\lesssim \,{\varepsilon }_{\text{B32}}\,\,\,\text{, valid for both equivalent strain measures (}{\varepsilon }_{v.M.}\,\text{or}\,{\varepsilon }_{Pmax}\text{)}\end{array}$$

The obtained difference Δ is below the range of the magnitude of Δ is nearly independent of *B*. The obtained variation for *B* is in the range of the computing precision. The influence of *f* is not definite. For practical considerations in future prototype studies, the difference between both Timoshenko beams is negligible, especially with regard to other errors such as the one of the skeletonisation step. It is interesting that the difference increases for greater *E*_*M*_. One possible reason is that the stiffness requirement between matrix and fibres (Eq. 2) is better fulfilled for lesser *E*_*M*_. For numerical considerations, a further investigation of this model aspect through additional sample geometries will be highly worthwhile.

#### Spatial distribution

The spatial distribution of matrix strain (plotted on a 5^2^-grid and averaged along the z-axis) shows a comparable pattern between the three samples including at the same time local variations (Fig. 5b). The main finding is that the matrix strain magnitude is not uniform across the samples under magnetic actuation. For uniaxial mechanical actuation, the samples show a very uniform deformation^12^. Under magnetic actuation in this present study, the minimum matrix strain can be found in the proximity of the kinematically constrained cube face *F*_*x*_ at *min*(*x*). Matrix strain magnitude increases with proximity to the non-constrained cube faces. In each of the samples, a maximum strain peak is located at the grid cell of *max*(*x*) and *max*(*y*). Inside the sample *V*, maxima of matrix strain exist too in each case. In the case of Sample-15%, a region of greatest strain on the cube’s top face visible in the plots of Fig. 4 corresponds to the local maximum in Fig. 5b. Consideration of the results in Fig. 5b will be mandatory for future prototype designs. The strain magnitude and its location clearly depend on the applied BC and local variations exist. The design of BC imposed on the assembly will have to be considered in future studies. This is found to be a novel and reproducible feature of this specific material.

#### Statistical distribution

The statistical distribution function *p*_(*x*)_ of *ε*_*v*.*M*._ is plotted in Fig. 5c and exhibits in each sample a single global *p*_(*x*)_-peak. This distribution pattern is obtained for each of the three samples. A change from *B* = 0.25 to 1.00 shifts this single global *p*_(*x*)_-peak towards greater values of *ε*_*v*.*M*._. This shift demonstrates that for greater *B* the matrix strain increases uniformly inside the sample. Conversely, greater *E*_*M*_ shifts the global *p*_(*x*)_-peak towards smaller values of *ε*_*v*.*M*._. The magnitude of *ε*_*v*.*M*._ ≈ 0.001 required for bone growth stimulation^38^ can be found locally in each sample for *B* = 0.25 and 1. This finding is mandatory for the intended application as tissue engineering scaffold. A localization of strain magnitude in the sample volume follows in the next section below.

### Matrix strain relative to fibre network and CP

The by far most relevant findings for the intended application purpose of this present study are shown in Fig. 6. Based on the strain distribution functions *g*_(*R*)_ and *f*_(*R*)_ (Eqs 12 and 13), it is possible to quantify and to analytically localize areas of greater or lesser matrix strain magnitude inside the material.

Figure 6a plots *f*_(*R*)_ which is the average matrix strain in *O*_(*n*,*R*)_ depending on *R* averaged for all network nodes *n* of the sample. The obtained results show that *R* has a strong influence on *f*_(*R*)_. A specific pattern is discovered independently of *f*, *E*_*M*_, or *B*. In the close proximity to the fibre structure (*R* → 0^+^), *f*_(*R*)_ exhibits in each sample one global peak which exceeds the sample average by several magnitudes. For increasing *R* ∈ [200 μm, 800 μm], *f*_(*R*)_ drops below the sample average. In this range, *f*_(*R*)_ is under the influence of the low-strain regions inside the samples and along *F*_*x*_ (Fig. 5b). For values of *R* > 800 μm, the regions of greater matrix strain near the non-constrained cube faces influence *f*_(*R*)_ stronger and it tends towards the sample average. This finding is particularly interesting for future research. One topic will be the mechanical interaction between fibres and the matrix at the interphase of these two. Biomechanical materials as used in the potential application of tissue engineering are characterized by non-linear mechanical response and additional parameters such as limited adhesion forces^64^. Those effects will be particularly important at the interface. Whether and how the predicted strain field changes remains to be seen.

The plots of Fig. 6b show *g*_(*R*)_, the matrix strain obtained on a sphere *o*_(*n*,*R*)_ according to the averaging method Eq. 12. The obtained matrix strain increases for greater *R* and a local maximum is located at around *R* = 600 μm. This pattern is obtained for each matrix material in each sample. The individual differences of network geometry and material density in the samples do not change this overall pattern. The absolute maximum of strain is obtained in the non-constrained sample corners at *R* ≈ 900 μm for Sample-10% and Sample-20%. In the case of Sample-15%, the sample maximum is reached at *R* ≈ 850 μm. In each of the three samples, the obtained matrix strain drops afterwards. One difference between the samples is that this drop is much more pronounced in the case of Sample-15%. Low magnitude of matrix strain in the non-constrained corners of this sample is visible in the deformation plots (Fig. 4). It is worth mentioning that at this value of *R* only a fraction of the total 10^6^ matrix hexahedra are located. The intersection of *o*_(*n*,*R*)_ with *V* for *R* > 850 μm in the sample corners affects only ≈2% of all matrix hexahedra. This causes the obtained differences which are statistically not representative.

Based on the results of Fig. 6b, it is possible to derive for the first time a simple analytical model for the prediction of local matrix strain inside the sample’s *V*. Eq. 16 approximates the obtained values by a power function:

16 $$\begin{array}{c}{\varepsilon }_{(R)}=aB{e}^{bR}\\ a\,[{{\rm{T}}}^{-1}],\,b\,[{{\rm{\mu }}{\rm{m}}}^{-1}]\in {{\mathbb{R}}}^{+}\end{array}$$

For *R* > 860 μm, the fit between Eq. 16 and the data declines sharply. This is why the regression analysis is run for *R* ≤ 860.25 μm. This means that 9,724 of 10^6^ hexahedra are not included in Eq. 16, ≈1%. The matrix strain of the other 99% is modelled by Eq. 16. Table 5 contains the regression values for *a*, *b*, and the coefficient of determination *r* ^2^. The values are given for *ε*_*v*.*M*._, *ε*_*Pmax*_, and for both Timoshenko beams. *r* ^2^ varies between 0.82 and 0.94 which indicates a very good fit. The values of *a* and *b* are very similar in each case for the two Timoshenko beams. A clear trend of *a* and *b* for changes of *f* can’t be identified at this stage. Whether this is achievable for greater *V* is one highly interesting topic for future work. A clear result of this study is that the strain magnitude exhibits a specific pattern around *CP* and that it can be described by this simple analytical model. Differences can be seen between the three samples. The overall pattern repeats.

**Table 5:** Regression analysis.

		von-Mises strain *ε*_*υ*.M._			max. principal strain *ε*_*Pmax*_		
Collagenous bone		*a* [T^−1^]	*b* [μm^−1^]	*r* ^2^ [−]	*a* [T^−1^]	*b* [μm^−1^]	*r* ^2^ [−]
Sample-10% for	B31	9.4169 · 10^−4^	6.7782 · 10^−3^	0.90	5.9554 · 10^−4^	7.1256 · 10^−3^	0.91
	B32	9.4177 · 10^−4^	6.7781 · 10^−3^	0.90	5.9565 · 10^−4^	7.1254 · 10^−3^	0.91
Sample-15% for	B31	7.3947 · 10^−4^	7.7463 · 10^−3^	0.83	6.4099 · 10^−4^	7.6340 · 10^−3^	0.83
	B32	7.3947 · 10^−4^	7.7463 · 10^−3^	0.83	6.4102 · 10^−4^	7.6340 · 10^−3^	0.83
Sample-20% for	B31	5.6691 · 10^−3^	5.7037 · 10^−3^	0.83	3.8894 · 10^−3^	5.9498 · 10^−3^	0.83
	B32	5.6691 · 10^−3^	5.7037 · 10^−3^	0.83	3.8894 · 10^−3^	5.9498 · 10^−3^	0.83
Granulation tissue		*a* [T^−1^]	*b* [μm^−1^ **]**	***r*** ^**2**^ **[−]**	***a*** [T^−1^]	***b*** [μm^−1^]	***r*** ^**2**^ [−]
Sample-10% for	B31	4.5103 · 10^−4^	5.6009 · 10^−3^	0.89	3.7185 · 10^−4^	5.5595 · 10^−3^	0.89
	B32	4.5118 · 10^−4^	5.6005 · 10^−3^	0.89	3.7199 · 10^−4^	5.5589 · 10^−3^	0.89
Sample-15% for	B31	1.4063 · 10^−4^	7.8491 · 10^−3^	0.83	1.2785 · 10^−4^	7.6628 · 10^−3^	0.83
	B32	1.4068 · 10^−4^	7.8486 · 10^−3^	0.83	1.2791 · 10^−4^	7.6621 · 10^−3^	0.83
Sample-20% for	B31	1.1270 · 10^−3^	5.7194 · 10^−3^	0.83	7.7026 · 10^−4^	5.9713 · 10^−3^	0.83
	B32	1.1270 · 10^−3^	5.7194 · 10^−3^	0.83	7.7025 · 10^−4^	5.9714 · 10^−3^	0.83
Immature bone		***a*** [T^−1^]	***b*** [μm^−1^]	***r*** ^**2**^ [−]	***a*** [T^−1^]	***b*** [μm^−1^]	***r*** ^**2**^ [−]
Sample-10% for	B31	3.9096 · 10^−5^	3.3689 · 10^−3^	0.91	2.5402 · 10^−5^	3.7762 · 10^−3^	0.94
	B32	3.9130 · 10^−5^	3.3679 · 10^−3^	0.91	2.5441 · 10^−5^	3.7740 · 10^−3^	0.94
Sample-15% for	B31	2.4758 · 10^−5^	4.6154 · 10^−3^	0.84	2.4109 · 10^−5^	4.3577 · 10^−3^	0.82
	B32	2.4861 · 10^−5^	4.6103 · 10^−3^	0.84	2.4194 · 10^−5^	4.3536 · 10^−3^	0.82
Sample-20% for	B31	2.3319 · 10^−5^	5.2677 · 10^−3^	0.90	1.9978 · 10^−5^	5.1900 · 10^−3^	0.90
	B32	2.3427 · 10^−5^	5.2618 · 10^−3^	0.90	2.0082 · 10^−5^	5.1833 · 10^−3^	0.90

*ε*_(*R*)_ = *aBe* ^*bR*^ (Eq. 16).

## Discussion

We have presented above the results for the deformation of a fibre-matrix composite under magnetic actuation. The local matrix strain has been studied and quantified in depth for the first time. The results show for the investigated assembly distinct deformation patterns under magnetic actuation and conclusions about the influence of model parameters can be drawn. With regard to the general material response, it must be taken from now on into consideration that the matrix deformation is not uniform across the samples under magnetic actuation. This is in contrast to previous work which has been able to show that this particular network material exhibits under uniaxial mechanical actuation a very uniform deformation^12^.

One additional conclusion from this study is that BC have a direct influence on the resulting deformation field under magnetic actuation. For the cube shaped samples in this study, the local matrix strain reaches its minimum in the proximity of the constrained cube face. Local maxima exist inside the sample volume *V*. Maxima are located in the free corners of the sample cubes. In future studies, the applied BC will have to be explicitly considered. One major material parameter is the fibre volume fraction *f*. It can be concluded for this model parameter that the magnitude of matrix strain increases for greater *f*. Whether this trend will also be obtained for other sample volume *V* is one very interesting topic for future research. A pronounced size effect of the mechanical response is known for this network material^12^.

The statistical distribution contains in each sample one single global peak which shifts depending on matrix stiffness and magnetic induction. The conclusion is that controlling the strain magnitude of this incidence peak is mandatory for controlling the average and overall response in possible future applications. At the same time, also local sections of greater or lesser strain magnitude exist inside the matrix. The results for two derived distribution functions (Eqs 12 and 13) allow to gain novel insights about the localization of matrix strain magnitude inside the material. First, it can be shown that the matrix strain increases in each sample in the proximity of the fibre structure. Second, the distance to the kinematically constrained cube face has a strong influence on matrix strain magnitude and can be used for deriving a simple analytical model of matrix strain magnitude (Eq. 16). This model is valid for 99% of the matrix volume. The achieved fit for the regression analysis is a very good match with *r* ^2^ = 0.82 or greater. A remaining challenge are the free, unconstrained corners of the cube.

With regard to the purpose of this study to investigate the suitability of the fibre-matrix composite for possible applications as bioactive scaffold in tissue engineering, one very important result is obtained. From experimental work, it has been known since 1985 that a cyclical strain of 0.001 of the frequency 1 Hz is beneficial for the stimulation of bone growth^38^. This strain magnitude must be considered as design target for the scaffold application. The results in this study are showing that this magnitude is achievable by this investigated fibre-network composite assembly. In conclusion, it is possible to say that the principal suitability of this material for bone growth stimulation under magnetic actuation is confirmed. Future work will have to relax the simplifications made for the matrix model. For the application as tissue engineering scaffold, understanding of local strain at the interface between cells and steel is mandatory. The advanced version of this current model could integrate biological aspects such as the detaching of focal adhesion points build by bone cells on the fibre structure^64^. Also a dynamic matrix model including the active migration of bone cells through the fibre network^65^ after the cell seeding would be a worthwhile aspect and of great interest for understanding bone development. The current model still relies on a static mesh.

In future work, one further effect must not be neglected in the evaluation of bone growth. It is known that magnetic fields alone already can stimulate bone growth. It is applied in the treatment of avascular necrosis or pseudarthrosis^39^. Designs for hip joint prostheses make use of that effect^40^. If benefits are achieved by the proposed assembly it will have to be made clear what the actual cause of the improvement is. The strain field imposed by the scaffold or the direct reaction of the tissue to a simple magnetic field? In the ideal case, it will be possible to raise synergies from both effects.
